# Acute postoperative pain management protocols in podiatric surgery within Australia: a Delphi study

**DOI:** 10.1186/s13047-022-00535-6

**Published:** 2022-04-11

**Authors:** Ping Ping Joanne Ang, Burke Hugo, Renee Silvester

**Affiliations:** The University of Western Australia, 35 Stirling Hwy, Crawley, WA 6009 Australia

**Keywords:** Postoperative pain management, Foot, Ankle, Foot and ankle surgery, Podiatric surgery, Delphi study

## Abstract

**Background:**

There is limited evidence in the literature to describe an analgesic protocol that takes into consideration the extent of foot and ankle surgery. The aim of this study was to develop a guide for acute postoperative pain management for podiatric surgery in Australia, and to identify opportunities to improve the current list of scheduled medicines available to podiatric surgeons.

**Methods:**

A Delphi method involving 3 survey rounds was employed for this study. Twelve expert panellists in the field of podiatric surgery and anaesthesiology were invited to participate, and 10 panellists remained by the end of the study. Round 1 involved 15 open-ended questions. These answers formed the basis of the 55 statements that were developed for the following 2 survey rounds, where panellists rated the appropriateness of each statement on a 9-point Likert scale. The third survey round was an opportunity for panellists to revise their answers to each statement in light of the majority response.

**Results:**

For mild acute postoperative pain, non-opioid oral analgesics were recommended as an appropriate management option. For moderate and severe acute postoperative pain, both non-opioid and opioid products were found to be appropriate by the majority. It was agreed that oral opioids be reserved for breakthrough pain at all severity levels. All other statements in the Delphi study pertaining to drug hypersensitivities or allergies, stratification of pain management, opioid prescription concerns, and access to pain medications were accepted as appropriate by the majority of panellists.

**Conclusion:**

The agreed approach to acute postoperative pain management for podiatric surgeons in Australia was with a stepwise approach, utilising multimodal therapy, and reserving oral opioids for breakthrough pain. Additionally, there was consensus for podiatric surgeons in Australia to have wider access to alternative analgesics and anti-emetics that have similar or improved efficacies with better safety profiles.

## Background

Pain is defined as a subjective unpleasant sensory and emotional experience associated with actual or potential tissue damage, or described in terms of such damage [1–3]. Postoperative pain stems from various causes such as neuronal damage, haematoma and oedema, infection, stiff joints, and intraoperative trauma [4]. Acute postoperative pain has been associated with an unacceptable negative affect on a patient’s quality of life, sleep and physical function, delayed return to normal daily activities, an increase in the postoperative hospital burden and economic costs due to re-admission, and potential development of chronic pain [2, 5, 6]. Orthopaedic patients have been found to report the highest incidence of pain compared with other types of surgical procedures, with more than half having suboptimal control [7–9]. Effective postoperative pain management should aim to minimise discomfort, facilitate early mobilisation and functional recovery, and prevent acute pain from developing into chronic pain [2, 6].

There is a vast amount of literature pertaining to strategies for acute postoperative pain management, with a recent upward trend into investigations for procedure specific protocols. The reason for this is manifold; the repercussions of surgically induced pain can delay healing and normal return to activities, prolong hospital stay, cause emotional distress, prompt fear and avoidance of surgery, and negatively impact a patient’s overall quality of life [7, 10–13]. Current advice for postoperative pain management includes general guidelines and multimodal approaches for acute pain management [10]. That said, there is recognition that such information should be procedure-specific to better tailor the analgesic efficacy of drugs between various types of surgery. Optimal dynamic pain relief has been found to be important for accelerated postoperative rehabilitation and minimising the unwanted side effects of certain drug classes on particular surgeries, as well as to potentially reduce the incidence of opioid-associated adverse effects with the use of non-opioid analgesics [14]. While a substantial portion of this area of orthopaedic-related research has focussed on postoperative pain management for larger surgeries such as total hip and knee arthroplasty, there is a paucity of evidence to guide pain management for foot and ankle surgery [5, 12, 15].

There have been suggestions in the literature for pain management strategies after elective foot and ankle surgery, however, these recommendations are dated and apply to practitioners living outside of Australia. Moreover, there is little evidence-based literature describing an analgesic protocol that factors in the spectrum of foot and ankle surgery. It is important for practitioners in Australia to have country-specific guidelines on prescribing strategies for acute postoperative pain management, given the differences between a practitioner’s access to certain analgesic drugs across the globe. The purpose of this study was to address this gap in the literature and develop a guide for acute postoperative pain management for podiatric day surgery in Australia. This research also aimed to identify opportunities to improve the current list of scheduled medicines available to podiatric surgeons in Australia and reach a consensus on the best practice for acute postoperative pain management. Meyr and colleagues stated that while a clinical consensus statement does not represent clinical practice guidelines, formal evidence reviews, recommendations, or evidence-based guidelines, in the scenario where high-level evidence is not always available, a clinical consensus statement reflects information from a group of experts based on the best available evidence, clinical experience, and common sense [12].

## Methods

The Delphi method was chosen for the study design as it is a suitable means for building consensus on a specific topic [16–18]. The Delphi process is typically conducted over a series of two or more sequential surveys known as “rounds”. While a varied number of rounds have been suggested for the Delphi process in the literature, 3 rounds were employed in this study as this number of iterations has been proposed to be sufficient for collecting the required data to reach a consensus in most scenarios [16]. It has been suggested multiple iterations through a series of surveys allows and encourages panellists to re-assess and have the option to amend their initial judgements about information provided in the previous rounds [16, 17].

The surveys in a Delphi study draw from a knowledgeable participant pool known as the “panellists” [16, 17]. Twelve panellists who met the inclusion criteria of being a podiatric surgeon with at least 3 years of experience prescribing medications or an anaesthetic consultant with pain management experience in podiatric or orthopaedic foot and ankle surgery were invited to participate in this study. No patients participated directly in this study. These panellists were recruited from their respective professional bodies including the Australian Association of Podiatric Surgeons (AAPS) and Australasian College of Podiatric Surgeons (ACPS), from private hospitals, as well as via the “snowball sampling” technique whereby panellists who had already been invited provided nominations for other candidates who also met the inclusion criteria. Given the niche area of podiatric surgery in Australia, snowball sampling was the most viable option to recruit from an already small pool of potential panellists, and offered accessibility to anaesthetic consultants who may work outside of the private sector where Australian podiatric surgeons solely practice. It was an assumption of this study that a participant who meets the inclusion criteria has sufficient clinical experience to provide thoughtful independent contribution regardless of their historical podiatric surgical training. Identification of a participant’s training may compromise the anonymity of their participation. It was therefore not of interest to collect this data nor assume its relevance for obtaining an agreed consensus for the use of medications in the acute postoperative setting.

The first round commenced with an open-ended questionnaire consisting of 15 questions that covered several topics on acute postoperative pain management after podiatric surgery in Australia. Conception of the initial open-ended survey was driven by the investigators who have clinical experience with prescribing for acute postoperative pain following podiatric surgery, including a practised podiatric surgeon with 8 years of prescribing, and a podiatric surgery registrar with 2 years of prescribing. After scoping the literature, the investigators identified that there was limited evidence to guide acute pain management after elective foot and ankle surgery in Australia. As alluded to earlier, at the time of conception, the evidence available related to practitioners outside of Australia, who have different access to certain analgesic drugs. Hence, as this Delphi study was a novel investigation with no reference point, the content of the initial open-ended survey began with broader questions that attempted to cover postoperative analgesic prescribing for the entire spectrum of foot and ankle surgery, to then synthesising a list of essential analgesics and/or a prescription plan for acute postoperative pain management in Australia based on panellist responses.

For the second survey round, a series of 55 statements was then developed based on information provided by the panellists from the first round. The questions were synthesised directly from answer provided, and any similar responses were grouped into one statement. The authors opted to present the panellists’ use of each proposed analgesic drug according to their patient’s pain severity levels. The pain severity levels were categorised as mild, moderate, and severe. Each category was defined according to an 11-point numeric rating scale (NRS) as follows: no pain was an NRS of 0 out of 10, while mild, moderate, and severe levels were an NRS of 1 to 3 out of 10, 4 to 7 out of 10, and 8 to 10 out of 10 respectively [19, 20]. Panellists were invited to rate the appropriateness of each of the 55 statements from 1 (extremely inappropriate) to 9 (extremely appropriate) using a Likert scale, similar to that used in Meyr and colleagues’ clinical consensus study [12].

The responses from the second round were analysed and grouped from 1 to 3 (inappropriate), 4 to 6 (neither appropriate nor inappropriate), and 7 to 9 (appropriate). The final survey presented the same 55 statements to the panellists as the previous round, however, this time a percentage summary demonstrating the initial consensus was included, and panellists were afforded the opportunity to revise their responses in light of this new information. Individual responses were not included with each round due to the limitations of the online survey tool used. All responses from each round were kept anonymous except to the research investigator, to preserve the integrity of the data.

This study adopted the design used in the modified Delphi study by Meyr and colleagues, and consensus was considered when majority of the panellists agreed the statement [12]. The main statistics typically used to present the collective judgements of respondents are measures of central tendency and level of dispersion [21]. The data was cleaned and analysed using IBM SPSS to obtain basic descriptive statistics such as the mean, median, mode, and standard deviation. Demographic data was also collected for background information. Any incomplete surveys or participant drop outs were omitted from the final analyses.

Ethics approval was obtained from the University of Western Australia Human Research Ethics Committee prior to the commencement of participant recruitment (2021/ET000438). Written informed consent was obtained from all panellists before commencement of data collection using the online survey platform, Qualtrics. The study was conducted over a period of 11 weeks.

## Results

Twelve panellists initially consented to participate in this study, however, due to the nature of the multiple iterations associated with the Delphi technique, drop out was expected. Ten panellists remained after the final survey round for inclusion in the study, with 1 podiatric surgeon and 1 anaesthetic consultant dropping out from the study. These panel members consisted of podiatric surgeons with endorsements for scheduled medicines (*n* = 8) and anaesthetic consultants (*n* = 2) practicing within Australia. Clinical experience ranged from 4 years to 30 years (median, 9.5 years) prescribing in clinical practice.

The consensus results of all 55 statements that were proposed are shown in Tables 1, 2, 3, 4, 5, 6, 7 and 8, with the mode of the grouped agreement level demonstrating the majority vote for each Delphi statement. The general pattern of agreement regarding drug prescribing was that for mild and moderate acute postoperative pain, oral medications such as paracetamol, paracetamol sustained release (SR) 665 mg, and/or a cyclooxygenase-2 (COX-2) inhibitor were agreed on as an appropriate management option, although there was an equal number of votes that deemed paracetamol SR 665 mg as neither appropriate nor inappropriate for moderate pain. An oral non-selective non-steroidal anti-inflammatory drug (NSAID) was deemed neither appropriate nor inappropriate for acute postoperative pain management at the mild and moderate pain severity levels. In addition to these drugs, an oral opioid combination product and/or oral tapentadol immediate release (IR) was also agreed as appropriate for management of moderate pain.

**Table 1:** General considerations – expert derived recommendations for round 2 and 3

Delphi Statement	Consensus^**a,b**^ (Median)		Interquartile range^**c**^	
	Round 2	Round 3	Round 2	Round 3
Perioperative prescribing for management of postoperative pain following podiatric surgery should be tailored based on extent of surgery and patient factors, not on anatomical location.	A (9)	A (9)	8.25–9	8–9
A stepwise approach should be taken depending on the extent of procedure, with an increase in combination of drugs (multimodal therapy) for larger procedures with more extensive dissection.	A (9)	A (9)	8–9	8.25–9

^a^The consensus represents the mode of the grouped agreement level (Inappropriate, 1–3; Neither appropriate nor inappropriate, 4–6; and Appropriate, 7–9)

^b^*A* Appropriate, *I* Inappropriate, *N* Neither appropriate nor inappropriate

^c^Interquartile range: Q1 – Q3

**Table 2:** Mild Pain (NRS 1–3 out of 10) – Expert derived recommendations for Round 2 and 3

Delphi Statement	Consensus^**a,b**^ (Median)		Interquartile range^**c**^	
	Round 2	Round 3	Round 2	Round 3
The following should be prescribed for mild postoperative pain after osseous and/or soft tissue type podiatric surgery:				
Oral paracetamol.	A (9)	A (8)	9–9	8.25–9
Oral paracetamol SR 665 mg.	A (8.5)	A (8)	7–9	7–9
An oral COX-2 inhibitor.	A (7)	A (8)	7–8	7.25–9
An oral non-selective NSAID.	N (5)	N (5)	4.25–7	5–6.75
An oral opioid combination product.	I, N (4)	I, N (5.5)	3–5	3–6
Oral oxycodone.	I (1.5)	I (2)	1–2	1–2.75
Oral tramadol.	I (1)	I (2)	1–3	2–2.75
Oral tapentadol IR.	I (1)	I (2)	1–3	1–2.75
Oral tapentadol SR.	I (1)	I (2)	1–1.75	1.25–2.75
Sublingual buprenorphine.	I (1)	I (2)	1–2	1–2.75
Postoperative oral opioids should be prescribed for a shorter duration for soft tissue procedures than osseous procedures.	A, N (5.5)	A (7)	5–7	4.25–8.5
Oral opioids such as paracetamol plus codeine, oxycodone, tapentadol, or tramadol should be prescribed for breakthrough pain.	A (7.5)	A (8)	7–9	8–9

^a^The consensus represents the mode of the grouped agreement level (Inappropriate, 1–3; Neither appropriate nor inappropriate, 4–6; and Appropriate, 7–9)

^b^*A* Appropriate, *I* Inappropriate, *N* Neither appropriate nor inappropriate

^c^Interquartile range: Q1 – Q3

**Table 3:** Moderate Pain (NRS 4–7 out of 10) – Expert derived recommendations for Round 2 and 3

Delphi Statement	Consensus^**a,b**^ (Median)		Interquartile range^**c**^	
	Round 2	Round 3	Round 2	Round 3
The following should be prescribed for mild postoperative pain after osseous and/or soft tissue type podiatric surgery:				
Oral paracetamol.	A (8)	A (7)	5.25–9	5.25–9
Oral paracetamol SR 665 mg.	A (7)	A, N (6.5)	5–8.75	5.25–8.75
An oral COX-2 inhibitor.	A (9)	A (9)	8.25–9	8–9
An oral non-selective NSAID.	N (5)	N (6)	4.25–6.75	5–7
An oral opioid combination product.	A (7)	A (7.5)	5.25–7	7–8.75
Oral oxycodone.	N (5)	N (6)	5–5.75	6–7.5
Oral tramadol.	N (5)	N (6)	5–6.75	5–7
Oral tapentadol IR.	A, N (6)	A (7)	5–7	6–7
Oral tapentadol SR.	N (5)	N (5.5)	4–6.5	4.25–7
Sublingual buprenorphine.	N (6)	N (4.5)	4–6.75	3–5.75
Postoperative oral opioids should be prescribed for a shorter duration for soft tissue procedures than osseous procedures.	A (6.5)	A (7)	5.25–8	5.25–7.75
Oral opioids such as paracetamol plus codeine, oxycodone, tapentadol, or tramadol should be prescribed for breakthrough pain.	A (8)	A (8)	7.25–8.75	7–9

^a^The consensus represents the mode of the grouped agreement level (Inappropriate, 1–3; Neither appropriate nor inappropriate, 4–6; and Appropriate, 7–9)

^b^*A* Appropriate, *I* Inappropriate, *N* Neither appropriate nor inappropriate

^c^Interquartile range: Q1 – Q3

**Table 4:** Severe Pain (NRS 8–10 out of 10) – Expert derived recommendations for Round 2 and 3

Delphi Statement	Consensus^**a,b**^ (Median)		Interquartile range^**c**^	
	Round 2	Round 3	Round 2	Round 3
The following should be prescribed for mild postoperative pain after osseous and/or soft tissue type podiatric surgery:				
Oral paracetamol.	A (6)	A (7)	3.5–9	3.25–9
Oral paracetamol SR 665 mg.	A (5)	N (6)	3.5–8.75	4.25–8.25
An oral COX-2 inhibitor.	A (9)	A (9)	7.5–9	7.25–9
An oral non-selective NSAID.	A, N (5.5)	A, N (6)	4.25–8.5	4.25–7
An oral opioid combination product.	A (8.5)	A, N (6.5)	7–9	5.25–7.75
Oral oxycodone.	A (8.5)	A (8)	8–9	7–8.75
Oral tramadol.	A (7.5)	A (7.5)	7–8	6.25–8.75
Oral tapentadol IR.	A (8.5)	A (8)	7.25–9	7.25–8.75
Oral tapentadol SR.	A (7.5)	A (7)	7–8	6.25–8
Sublingual buprenorphine.	A (8)	A (7)	5–8	3.25–7.75
Postoperative oral opioids should be prescribed for a shorter duration for soft tissue procedures than osseous procedures.	A (7.5)	A (7)	5.25–8.75	5–7.75
Oral opioids such as paracetamol plus codeine, oxycodone, tapentadol, or tramadol should be prescribed for breakthrough pain.	A (9)	A (9)	8.25–9	9–9

^a^The consensus represents the mode of the grouped agreement level (Inappropriate, 1–3; Neither appropriate nor inappropriate, 4–6; and Appropriate, 7–9)

^b^*A* Appropriate, *I* Inappropriate, *N* Neither appropriate nor inappropriate

^c^Interquartile range: Q1 – Q3

**Table 5:** Drug Hypersensitivities / Allergies – Expert derived recommendations for Round 2 and 3

Delphi Statement	Consensus^**a,b**^ (Median)		Interquartile range^**c**^	
	Round 2	Round 3	Round 2	Round 3
If a patient is hypersensitive or allergic to an NSAID, paracetamol or opioids should be used.	A (8.5)	A (9)	8–9	8–9
If a patient is hypersensitive or allergic to an opioid, paracetamol or an NSAID should be titrated to tolerance.	A (8.5)	A (8.5)	8–9	8–9
If a patient is hypersensitive or allergic to a specific opioid, an alternative opioid should be used.	A (8.5)	A (8.5)	8–9	8–9
If a patient is hypersensitive or allergic to NSAIDs and opioids, paracetamol, gabapentinoids, or ketamine should be used.	A (8)	A (8)	6.25–9	7–8.75
A combination of long-acting local anaesthetic mixed with dexamethasone (or other corticosteroid) or intraoperative intravenous dexamethasone should be administered for patients with allergies to NSAIDs and/or opioids, or for general use in a preoperative or intraoperative setting.	A (8.5)	A (8)	8–9	7–9

^a^The consensus represents the mode of the grouped agreement level (Inappropriate, 1–3; Neither appropriate nor inappropriate, 4–6; and Appropriate, 7–9)

^b^*A* Appropriatem *I* Inappropriate, *N* Neither appropriate nor inappropriate

^c^Interquartile range: Q1 – Q3

**Table 6:** Opioid Prescription Concerns – Expert derived recommendations for Round 2 and 3

Delphi Statement	Consensus^**a,b**^ (Median)		Interquartile range^**c**^	
	Round 2	Round 3	Round 2	Round 3
Sedation and/or respiratory depression with opioid use is a concern given that there is a potential risk of this side effect.	A (7.5)	A (7.5)	6–8	7–8
There are concerns about the use of codeine due to its unpredictability with some patients being poor or ultra-rapid metabolisers.	A (8)	A (7)	7–8	7–7.75
There are concerns about the prescription of narcotics to the elderly age group in particular.	A (9)	A (7.5)	7.25–9	7–8
Overuse and prolonged use leading to addiction/dependence is a concern with the prescription of opioids for pain management following podiatric surgery.	A (6)	A (6.5)	5–8	3.5–7

^a^The consensus represents the mode of the grouped agreement level (Inappropriate, 1–3; Neither appropriate nor inappropriate, 4–6; and Appropriate, 7–9)

^b^*A* Appropriate, *I* Inappropriate, *N* Neither appropriate nor inappropriate

^c^Interquartile range: Q1 – Q3

**Table 7:** Access to Pain Medications in Australia – Expert derived recommendations for Round 2 and 3

Delphi Statement	Consensus^**a,b**^ (Median)		Interquartile range^**c**^	
	Round 2	Round 3	Round 2	Round 3
Currently for postoperative pain management following podiatric surgery, podiatric surgeons in Australia have access to over-the-counter medications, local anaesthetics, schedule 4 NSAIDs, injectable/topical corticosteroids, opioids (codeine and oxycodone in short acting and IR form), promethazine for anti-emetic purposes, and injectable naloxone.				
Podiatric surgeons in Australia should have access to a higher oral oxycodone dose strength and duration of course than the current regimen (i.e. Must only prescribe up to 10 mg doses, maximum of 20 mg in 24 h, for a maximum of 3 days.)	A (8)	A (9)	7–9	5.75–9
In addition to current the endorsements allowed for pain management listed above, podiatric surgeons in Australia should have prescription rights for other oral opioids such as tapentadol and/or tramadol.	A (9)	A (9)	9–9	9–9
In addition to current the endorsements allowed for pain management listed above, podiatric surgeons in Australia should have prescription rights for other opioids such as sublingual buprenorphine.	A (9)	A (9)	7.25–9	8–9
In addition to current the endorsements allowed for pain management listed above, podiatric surgeons in Australia should have prescription rights for other non-opioid oral alternatives such as gabapentin, pregabalin, and ketamine.	A (9)	A (9)	8–9	9–9
In addition to current the endorsements allowed for pain management listed above, podiatric surgeons should have access to other anti-emetic medications, for patients who experience nausea and vomiting.	A (9)	A (9)	9–9	9–9
Podiatric surgeons in Australia should have access to an open formulary, ensuring that further specialist opinion is sought to ensure optimal pain management.	A (9)	A (9)	9–9	8.25–9

^a^The consensus represents the mode of the grouped agreement level (Inappropriate, 1–3; Neither appropriate nor inappropriate, 4–6; and Appropriate, 7–9)

^b^*A* Appropriate, *I* Inappropriate, *N* Neither appropriate nor inappropriate

^c^Interquartile range: Q1 – Q3

**Table 8:** Stratification of Pain Management – Expert derived recommendations for Round 2 and 3

Delphi Statement	Consensus^**a,b**^ (Median)		Interquartile range^**c**^	
	Round 2	Round 3	Round 2	Round 3
A postoperative pain management plan following podiatric surgery should be based on type and/or extent of surgery, osseous involvement with or without internal fixation, and patient factors (i.e. age, comorbidities, or drug interactions/sensitivities), not on location of surgery.	A (9)	A (8.5)	8.25–9	8–9
Psychosocial factors and previous success or failure with certain analgesics should guide choice of drug and dosing regimen for postoperative pain management following podiatric surgery.	A (9)	A (9)	8–9	8.25–9

^a^The consensus represents the mode of the grouped agreement level (Inappropriate, 1–3; Neither appropriate nor inappropriate, 4–6; and Appropriate, 7–9)

^b^*A* Appropriate, *I* Inappropriate, *N* Neither appropriate nor inappropriate

^c^Interquartile range: Q1 – Q3

For severe acute postoperative pain, oral paracetamol and/or an oral COX-2 inhibitor, and oral formulations of oxycodone, tramadol, tapentadol IR and SR, and/or sublingual buprenorphine were voted as an appropriate management option. In this scenario, oral paracetamol SR 665 mg was considered neither appropriate nor inappropriate, while an oral NSAID or opioid combination product were tied in votes as appropriate and neither appropriate nor inappropriate. All other statements in the Delphi study were accepted as appropriate by the majority of panellists.

## Discussion

### General overview of findings

This study consolidated a general guide for acute postoperative pain management following podiatric day surgery in Australia based on the severity of pain. The majority of panellists agreed acute postoperative pain management for podiatric surgery should be based on type and/or extent of surgery, osseous involvement with or without internal fixation, and patient factors such as age, comorbidities, or drug interactions or sensitivities. Therefore, it was prudent to classify the prescribing approach according to pain severity as this would incorporate the various factors that influence pain. In general, the intensity of pain has been thought to correspond to the magnitude of surgical injury and type of surgery, with bony procedures being more painful than soft-tissue procedures, as the periosteum has the lowest pain threshold of the deep somatic structures [14, 22]. Hence, it can be expected that more involved soft tissue dissection and osseous surgery corresponded with increased pain levels.

The main findings of this study were in line with the general recommendations found in the literature. While the exact combination of analgesic drugs used could not be determined from this survey due to the numerous combinations possible, the panellists’ responses demonstrated a pattern that reflected a stepwise approach to pain management, with an escalation to stronger analgesics with increasing pain levels, as well as a recommendation for utilising multimodal therapy for these larger procedures that require more extensive dissection. There was also majority view that oral opioids such as paracetamol plus codeine, oxycodone, tapentadol, or tramadol should be reserved for breakthrough pain at all pain severity levels (Tables 2, 3 and 4). A combination of long-acting local anaesthetic mixed with a corticosteroid like dexamethasone, was agreed on for general use in a preoperative or intraoperative setting (Table 5).

### Multimodal analgesia for acute postoperative pain

It is widely accepted that acute postoperative pain management should employ a multimodal approach, but there is little consensus with regards to the specific combinations of analgesics used in specific situations [12]. Multimodal treatment has been shown to provide superior pain relief, and a reduction in opioid dependence and opioid related adverse events [5, 7, 12, 23, 24]. The combined drug synergy effects that block generation and perception of pain at several different stages in the pain pathway has been reported to be necessary for effective pain control [7, 12]. Several studies have reported on optimal pain management strategies through multimodal analgesia for elective foot and ankle surgeries with the use of the paracetamol, NSAIDs, opioids, and α2δ-ligands, and regional anaesthesia [5, 7, 12]. There was also indication from this study that multimodal therapy should be adopted for acute postoperative pain management, however, this could not be fleshed out further than identifying the most commonly accepted and prescribed analgesics. This was in part due to the large number of potential drug combinations being unfeasible to include in a single survey round.

### Stepwise approach to acute postoperative pain management

**Fig. 1:**
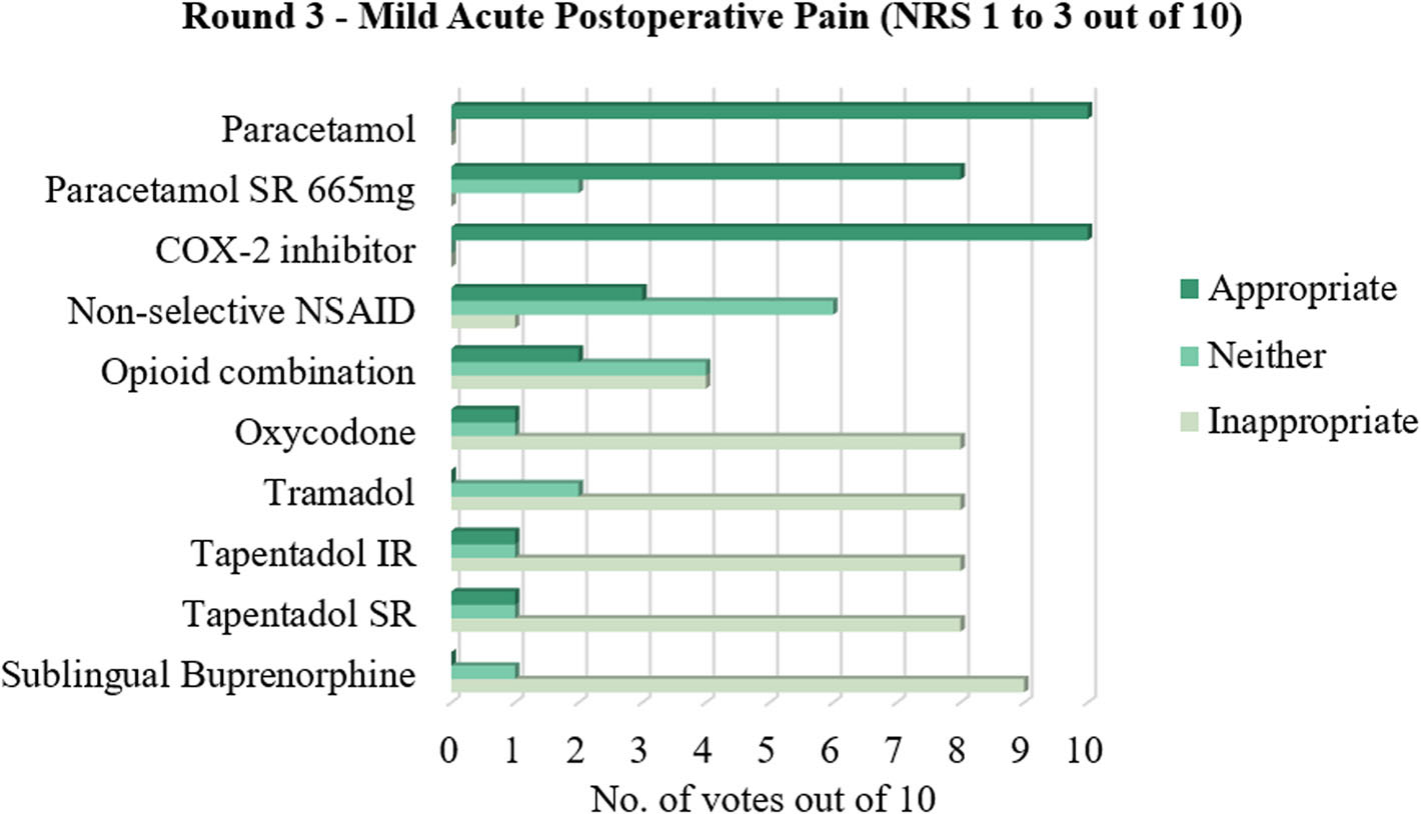
Number of votes for each analgesic for mild acute postoperative pain following podiatric surgery

**Fig. 2:**
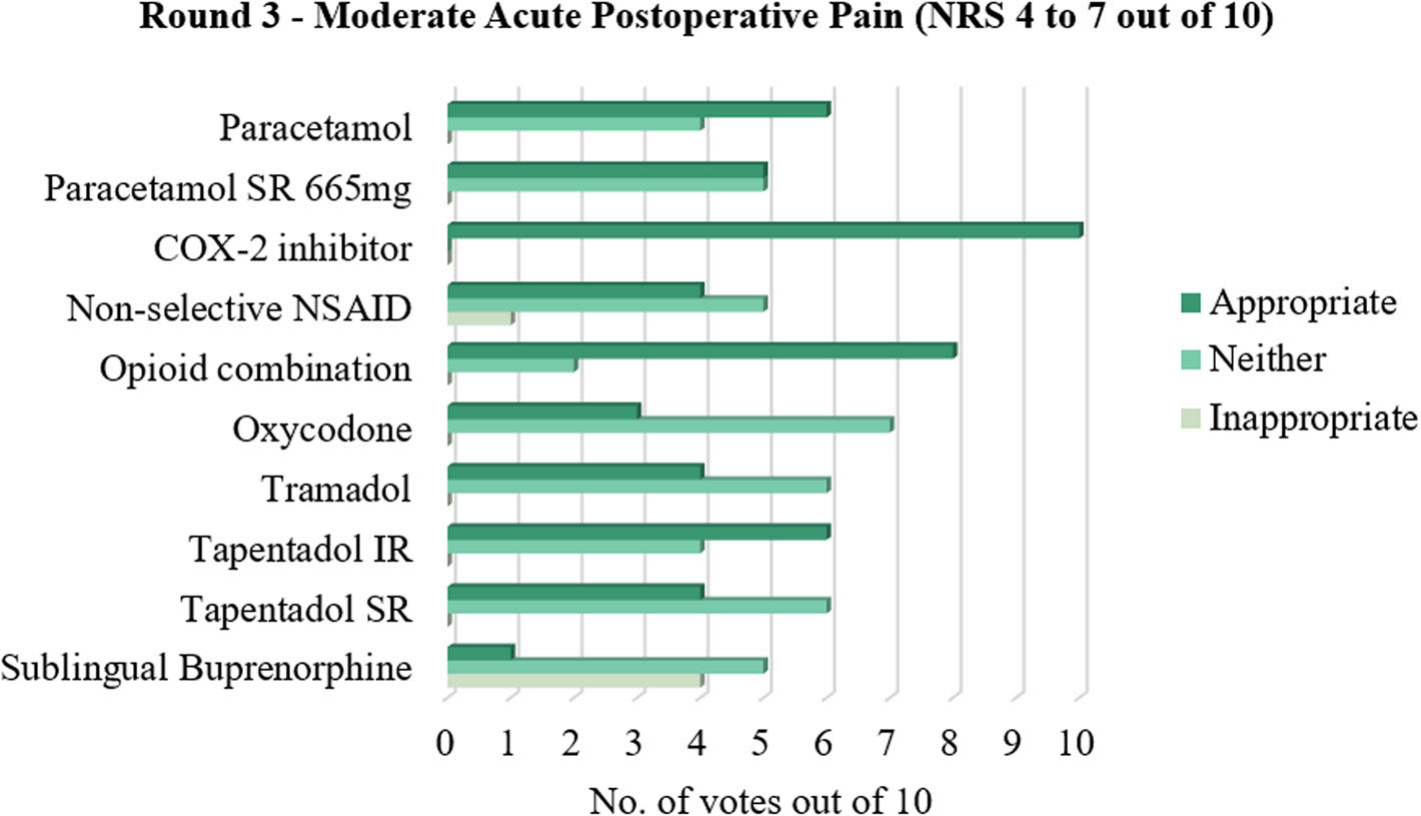
Number of votes for each analgesic for moderate acute postoperative pain following podiatric surgery

**Fig. 3:**
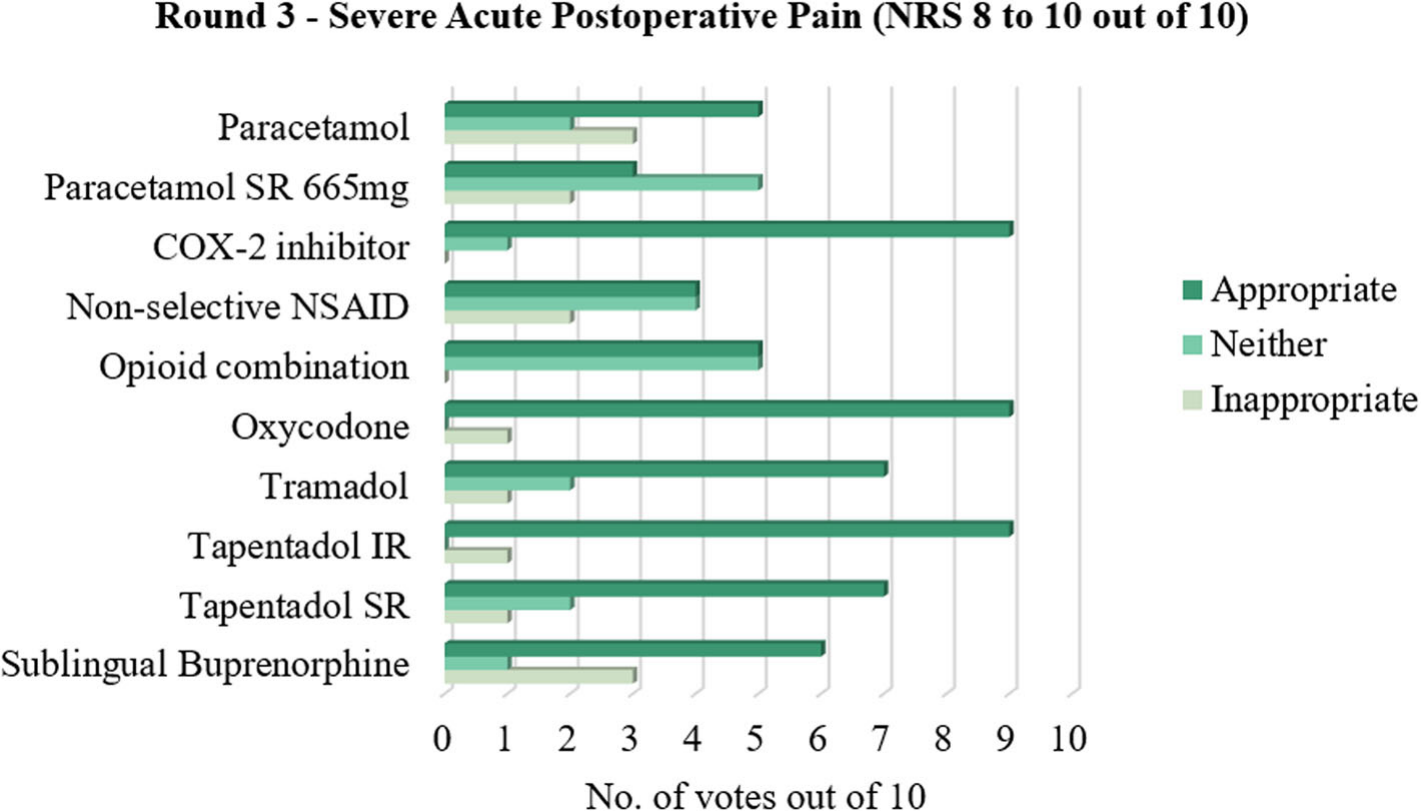
Number of votes for each analgesic for severe acute postoperative pain following podiatric surgery

This study supports a stepwise approach to acute postoperative pain management after podiatric surgery. A majority of panellists voted in favour of non-opioids and against opioids for mild acute postoperative pain (Fig. 1). This suggests paracetamol, paracetamol SR, and/or a COX-2 inhibitor be prescribed as first-line treatment for mild acute postoperative pain, while a non-selective NSAID and opioid combination product may be used with discretion. For moderate acute postoperative pain, paracetamol, a COX-2 inhibitor, opioid combination product, and/or tapentadol IR were suggested as the first-line options, while the other drug options were found to be neither appropriate nor inappropriate (Fig. 2). This could be attributed to the fact that moderate pain is a middle ground where titrating the right amount of analgesia can be difficult; that is, inadequate analgesia could negatively impact the patient’s pain experience and recovery, while overprescribing increases the risk of the patient experiencing unwanted adverse effects. For severe acute postoperative pain, this study suggested paracetamol, a COX-2 inhibitor, and oral opioids such as combination products, oxycodone, tramadol, tapentadol, or sublingual buprenorphine should be considered as first-line treatment, with greater consensus for opioids at this level of pain (Fig. 3).

It should be clarified that the panellists are not recommending that patients are prescribed all of the medications deemed appropriate for use at the specified pain levels, but rather that a selection of these medications that are deemed appropriate should be made while taking into account safe doses, a patient’s existing list of medications, as well as other patient factors.

### Opioids for breakthrough pain

Opioids have often been reported to be used as rescue analgesics used at higher levels of pain or for breakthrough pain. This study results recommend oral opioids such as codeine, oxycodone, tapentadol, or tramadol be reserved for breakthrough pain at all levels. While these drugs may be considered inappropriate for milder pain levels, they should still be considered when necessary, and conversely, while these drugs may be deemed appropriate for more severe pain, they may not necessarily be required. This mirrors the view in the literature that opioids be used as rescue analgesics and only as required due to their potential undesirable effects that could impede recovery [5]. It should also be noted that at all pain levels in this study, a COX-2 inhibitor was found to be highly appropriate, while a non-selective NSAID was generally found to be neither appropriate nor inappropriate.

### Non-selective NSAIDs and COX-2 inhibitors

NSAIDs are commonly used as first-line medications to treat postoperative pain and inflammation. Wang and colleagues proposed the combination of paracetamol and conventional NSAID or COX-2 inhibitors should be used whenever possible [5]. Contrary to this, Kohring and Orgain recommended non-selective NSAIDs and COX-2 inhibitors be used for postoperative pain management after soft tissue procedures, but not for procedures that require bone healing [7]. There is level III evidence to corroborate that short-term, low-dose use of NSAIDs and COX-2 inhibitors may have an inhibitory effect on bony healing, but not on soft tissue healing [7, 25]. Other studies on the effects of NSAIDs on bone healing concluded that in the absence of robust clinical or scientific evidence, clinicians should treat NSAIDs as a risk factor for bone healing impairment, and their administration should be avoided in high risk patients [26, 27].

The results from this study align more closely with that of Wang and colleagues. It was agreed an oral COX-2 inhibitor was appropriate for acute postoperative pain management at all pain levels by an overwhelming majority of the panellists. This could be attributed to the fact that inhibition of COX-1 results in platelet dysfunction and gastrointestinal toxicity, and COX-2 inhibitors have little or no effect on COX-1 at therapeutic doses, thus providing anti-inflammatory and analgesic actions with a reduced risk of these unwanted COX-1 side effects [7, 28]. It should be noted, however, that COX-2 inhibitors are still associated with adverse effects, including but not limited to gastrointestinal adverse effects [28]. In contrast, a non-selective NSAID was found to be neither appropriate nor inappropriate by the majority in all but the severe pain level, where the vote was tied between being appropriate and neither appropriate nor inappropriate. While a non-selective NSAID may pose a greater risk to platelet and gastrointestinal function compared to COX-2 inhibitors, it still offers the benefits of analgesia, accessibility, low cost, and non-addictive properties that provide a suitable pain management option for the carefully chosen patient.

### Opioid prescribing practice and concerns

Prescribing opioids for acute pain management in foot and ankle surgery is highly varied and without formal recommendation. The literature has few guidelines for opioid-prescribing practices, with only anecdotal suggestions offered, and because physicians cannot accurately predict how much analgesia a patient will require, opioids tend to be overprescribed [9, 10, 15, 23, 29–31]. Similarly, this study had a lack of a definitive consensus on prescribing opioid analgesics for moderate acute postoperative pain, however, the consensus to use opioids at more severe pain levels in this study is reflective of the literature. These include a level II prospective observational cohort study that proposed patients who had hindfoot, ankle, or osseous procedures required more opioids [9], as well as the study by Chan et al. that found fewer opioids were required postoperatively for percutaneous foot and ankle surgery due to the smaller incisions and minimal disruption to surrounding soft tissue [31].

There was agreement from panellists that oral opioids should be prescribed for a shorter duration for soft tissue procedures compared to osseous procedures. This can most likely be attributed to the opioid prescription concerns raised by the majority of panellists (Table 6). The addictive potential and side-effect profile associated with opioids are widely recognised in the literature; consequently, the increase in accidental death and high health care costs reported with opioid abuse has created a need for alternative methods of pain control, as well as legislation restricting opioid prescriptions [7, 23, 29, 32].

### Scheduled medicines list for podiatric surgeons

The secondary aim of this study was to generate consensus on improvements to the current list of scheduled medicines available to podiatric surgeons in Australia, particularly for acute postoperative pain management. The panellists agree that podiatric surgeons in Australia should have access to a higher oral oxycodone dose strength and duration of course than the current allowance as outlined in Table 7. The results demonstrated the majority of panellists were advocates for access to other opioid alternatives such as oral tramadol, oral tapentadol, or sublingual buprenorphine; other non-opioid alternatives such as gabapentin, pregabalin, or ketamine; and additional anti-emetic medications.

One of the more contentious topics was the consensus for podiatric surgeons to have access to an open formulary with further specialist input, to achieve optimal pain management. The surgeon is logically the healthcare practitioner best equipped to understand the patient’s need for pain control given their close involvement throughout the patient’s surgical journey, while the pain specialists bring their in-depth knowledge and expertise on pain management to the table. Not only would this recommendation allow the surgeon to assume responsibility with continuity of care, at the same time it also promotes interdisciplinary involvement in acute postoperative pain management when other specialist input is obtained. This is advantageous to both patient and practitioner as the benefits of more immediate and complete facilitation of care with these safety precautions and tailored analgesia outweigh the risk of adverse events and potential delays with treatment. It is, however, imperative prescribers seeking to obtain access to a wider range of medications have appropriate training, so as to ensure the principles of safe prescribing are adhered to at all times.

### Psychosocial factors and previous success or failure

Finally, other factors beyond drug efficacy and side-effects need to be considered when prescribing for acute postoperative pain. Panellists recommended that psychosocial factors and previous success or failure with certain analgesics should guide choice of drug and dosing regimen (Table 8). A salient point made by Kohring and Orgain was that clinical judgement should always be applied to adjust, omit, or substitute appropriate medications, doses, intervals, and durations to ensure safe and optimal care [7].

### Strengths, limitations, and future studies

The strength of this Delphi study was that it afforded an avenue to guide group opinion from experts within their fields toward a final decision with the benefit of panellist anonymity, which has been proposed to reduce the effects of conformity to the dominant view and the bandwagon effect [16, 17]. Moreover, it was a useful platform to initiate discussion on topics of controversy or that have a lack of clarity, such as opioid prescribing and additional endorsement rights for podiatric surgeons.

Several limitations accompany the methodology and analysis of this study. Due to time constraints, creation of this study’s initial open-ended survey was limited to questions developed by the investigators, without the use of phone interviews and face-to-face conversations. One of the major limitations of this research method included a lack of a cut-off for consensus. The literature suggests variation in the level of consensus reported, with some studies defining a consensus level of 75 to 80%, while others omitted a defined cut-off [18]. Nevertheless, due to the explorative nature of the study, the authors felt that the results would be better captured by demonstrating the majority vote, instead of excluding statements that fail to meet an arbitrary cut-off. There was also the potential for researcher or subject bias and a lack of strong evidence to support its reliability [16, 21]. That is, if these same surveys were proposed to a different panel, there could be a possibility for different outcomes [16, 21]. Another potential weakness of this study is the participating panellists and their potential conflicts of interest; specifically, the number of participating podiatric surgeons outweighed the number of anaesthetists involved, and thus, could be considered to have skewed the results. While the results show the majority vote for each Delphi statement, they do not illustrate whether the anaesthetists concurred with the views of podiatric surgeons. The exclusion of direct and/or indirect patient contribution to this study could also be considered a limitation, given the era of patient centred care and shared decision making.

Goodman stated the final judgement should always consider the distribution of responses and be aware that the group’s agreement may not be as significant as it appears [17]. Future studies may consider a panellist group consisting of equal numbers of podiatric surgeons and anaesthetists, and clearly delineate an algorithm for the combination of drugs and their dosing regimen for acute postoperative pain management according to each severity level. The use of an independent researcher, who is not involved in the data analyses, to conduct focus groups and phone or face-to-face interviews to develop the first round survey questions, may improve the rigour of this type of mix-methods study.

## Conclusion

In summary, the agreed approach to acute postoperative pain management for podiatric surgeons in Australia was with a stepwise approach, utilising multimodal therapy, and reserving oral opioids for breakthrough pain. Additionally, there was consensus for podiatric surgeons in Australia to have wider access to alternative analgesics and anti-emetics that have similar or improved efficacies with better safety profiles.

## Data Availability

The datasets used and/or analysed during the current study are available from the corresponding author on reasonable request.

## Abbreviations

AAPS: Australian Association of Podiatric Surgeons
ACPS: Australasian College of Podiatric Surgeons
COX-2: Cyclooxygenase-2
IR: Immediate release
NSAID: Non-steroidal anti-inflammatory drug
SR: Sustained release
